# Effects of Long-Term Endogenous Corticosteroid Exposure on Brain Volume and Glial Cells in the AdKO Mouse

**DOI:** 10.3389/fnins.2021.604103

**Published:** 2021-02-10

**Authors:** Jorge Miguel Amaya, Ernst Suidgeest, Isabelle Sahut-Barnola, Typhanie Dumontet, Nathanaëlle Montanier, Guilhem Pagès, Cécile Keller, Louise van der Weerd, Alberto M. Pereira, Antoine Martinez, Onno C. Meijer

**Affiliations:** ^1^Department of Endocrinology, Leiden University Medical Center, Leiden, Netherlands; ^2^Department of Radiology, Leiden University Medical Center, Leiden, Netherlands; ^3^Génétique Reproduction et Développement, Université Clermont-Auvergne, CNRS, INSERM, Clermont-Ferrand, France; ^4^INRAE, AgroResonance, QuaPA UR370, Saint-Genès-Champanelle, France; ^5^Human Genetics Department, Leiden University Medical Center, Leiden, Netherlands

**Keywords:** glucocorticoid, glia, myelin basic protein, glial fibrillary acidic protein, ionized calcium binding adaptor molecule 1

## Abstract

Chronic exposure to high circulating levels of glucocorticoids has detrimental effects on health, including metabolic abnormalities, as exemplified in Cushing’s syndrome (CS). Magnetic resonance imaging (MRI) studies have found volumetric changes in gray and white matter of the brain in CS patients during the course of active disease, but also in remission. In order to explore this further, we performed MRI-based brain volumetric analyses in the AdKO mouse model for CS, which presents its key traits. AdKO mice had reduced relative volumes in several brain regions, including the corpus callosum and cortical areas. The medial amygdala, bed nucleus of the stria terminalis, and hypothalamus were increased in relative volume. Furthermore, we found a lower immunoreactivity of myelin basic protein (MBP, an oligodendrocyte marker) in several brain regions but a paradoxically increased MBP signal in the male cingulate cortex. We also observed a decrease in the expression of glial fibrillary acidic protein (GFAP, a marker for reactive astrocytes) and ionized calcium-binding adapter molecule 1 (IBA1, a marker for activated microglia) in the cingulate regions of the anterior corpus callosum and the hippocampus. We conclude that long-term hypercorticosteronemia induced brain region-specific changes that might include aberrant myelination and a degree of white matter damage, as both repair (GFAP) and immune (IBA1) responses are decreased. These findings suggest a cause for the changes observed in the brains of human patients and serve as a background for further exploration of their subcellular and molecular mechanisms.

## Introduction

Glucocorticoid hormones (GCs) are mediators of the response to stress, a state following real or perceived threat to homeostasis (Smith and Vale, 2006). GCs act throughout the body *via* the widely expressed glucocorticoid receptor (GR) and the mineralocorticoid receptor (MR) (Pujols et al., 2002). GC binding to its receptors results in a wide array of genomic and non-genomic signaling changes at the cellular level (Kadmiel and Cidlowski, 2013). A major aspect of GR-mediated effects concerns its metabolic effects on carbohydrate, lipid, and protein metabolism (from their action on liver, adipose tissue, and muscle), which in the long term predispose for obesity and metabolic syndrome. Chronic exposure to high levels of GCs increases visceral adipose tissue (Debono et al., 2013) and upregulates the expression of lipogenic pathway genes (Hochberg et al., 2015). Accordingly, central obesity and dyslipidemia are very common signs in Cushing’s syndrome (CS) patients, a disorder caused by prolonged exposure to excess GCs (Angeli et al., 1997; Garrapa et al., 2001; Sharma et al., 2015). By screening populations of patients with simple obesity, prevalence rates of CS might be as high as 9% (Tiryakioglu et al., 2010).

GCs also affect brain function. The effects on peripheral energy metabolism and immunity alone may already do this (Koorneef et al., 2018), but MR and GR are also widely (but differentially) expressed in neurons and other cell populations in the brain. Cortisol affects a wide range of brain processes, including food intake (mirroring peripheral effects on metabolism), cognition, emotion, and autonomic responses. The effects may involve biochemical and structural changes (de Kloet et al., 2005). The study of GC actions in the brain has served to illustrate two closely related concepts: (1) The effects of GC can be adaptive or deleterious, and (2) cellular responses to GCs are different depending on the duration of exposure (acute vs chronic) (McEwen et al., 2015). These two concepts are also illustrated clinically by changes in the brain of CS patients.

Cushing’s syndrome patients in active disease have been reported to present with smaller hippocampal volumes (Starkman et al., 1992), larger ventricular diameters, and cerebral atrophy (Simmons et al., 2000; Bourdeau et al., 2002). Strikingly, patients in long-term remission present smaller volumes in total gray matter (Resmini et al., 2012), particularly in the anterior cingulate cortex (Andela et al., 2013), as well as decreased cortical thickness (Crespo et al., 2014; Bauduin et al., 2020). Changes are not limited to gray matter though, as these patients also present significant reductions in white matter integrity (van der Werff et al., 2014).

Recently, we developed mouse models of endogenous CS: the AdKO mice, which carry an inactivating deletion of the gene encoding the regulatory subunit 1 alpha of the PKA, specifically targeted to the adrenal cortex. These mice present adrenal hyperplasia, chronic hypercorticosteronemia, lack of negative feedback after exogenous GCs, and even fat accumulation in the back of the neck (“buffalo hump”), which is one of the most visible signs in CS patients (Sahut-Barnola et al., 2010; Drelon et al., 2016; Dumontet et al., 2018). Here, we used brain magnetic resonance imaging (MRI) in order to find whether the changes observed in human patients were mirrored in our mice. We also performed immunohistochemical staining of glial cells in an attempt to explore the origins of such changes.

## Materials and Methods

### Mice

All animal work was conducted according to French and European directives for use and care of animals for research purposes and received approval from the French Ministry of Higher Education, Research and Innovation (APAFIS#21153-2019061912044646 v3). The *Sf1-Cre* (Cre expression in all steroidogenic cells of the adrenal cortex from its inception) (Bingham et al., 2006) and *Prkar1afl/f*l (floxed allele of *Prakar1a* that allows Cre-mediated inactivation of R1α subunit and subsequent constitutive activation of PKA catalytic activity; Kirschner et al., 2005) were used as breeders. Mice were all maintained and bred on a mixed background. Throughout the manuscript, AdKO_2.0_ refers to *Sf1-Cre*::*Prkar1afl/fl*, and WT refers to littermate control animals. ACTH-independent CS features of mice bearing R1α inactivation in the adrenal cortex using various Cre-expressing lines were previously described (Sahut-Barnola et al., 2010; Drelon et al., 2016; Dumontet et al., 2018). AdKO_2.0_ mice were used here because of their more severe CS phenotype compared to the original AdKO line that used the *Akr1b7* Cre driver (Sahut-Barnola et al., 2010). Mice from both sexes were analyzed at 8–10 weeks of age (*n* = 6 for males, *n* = 6 for females). Femur length was measured for a subset of the mice (*n* = 3 for male, *n* = 4 for females).

### Magnetic Resonance Imaging

Mice were anesthetized with 2% isoflurane and perfused for 1 min with 1 × phosphate-buffered saline (PBS) and for 4 min with 4% paraformaldehyde (PFA). The skull was freed from the skin and fat tissue and stored in 4% PFA overnight and then transferred to 4% PFA + 1:40 v/v gadoteric acid 0.5 mmol/ml (Dotarem, Guerbet, France) at 4°C for 3 weeks. Then they were transferred to a solution of 1 × PBS, 1:40 v/v Dotarem, and 0.01% sodium azide, and after 2 days, an MRI scan was acquired. MRI acquisitions were performed on a 9.4-T Bruker magnet (Bruker, Germany). The magnet was equipped with a 15-mm microimaging radiofrequency (RF) coil and maximum gradients up to 1.5 T m^–1^ along the three axes. The fixed brain was introduced inside a flat-bottom 15-mm NMR tube (Hilgenberg, Germany), and Fomblin^®^ (Sigma-Aldrich, France), a perfluorinated polyether, was added to limit the magnetic susceptibility at the interfaces without generating any NMR signal. 2D fast low-angle shot (FLASH) acquisition pilot scans were performed in three planes: axial, sagittal, and coronal. A whole-brain image was acquired based on a 3D-FLASH protocol. The echo and repetition times were set to 5.3 and 15.0 ms, respectively. The flip angle was 30° with a bandwidth of 3 kHz. The field of view (FOV), covering the whole brain, was 16 mm × 14 mm × 15 mm with a matrix of 320 × 280 × 300 points, leading to an isotropic resolution of 50 μm. Eight averages were performed, leading to an experimental time of around 2 h and 50 min. One MRI data set (WT male) was excluded from further MRI analysis due to the presence of imaging artifacts.

The MRI scans were linearly (six parameters followed by 12 parameters) and subsequently nonlinearly registered using a combination of mni_autoreg tools (Collins et al., 1994) and advanced normalization tools (ANTs; Avants et al., 2008, 2011), resulting in unbiased alignment of all scans. A population atlas was created by resampling all MRI scans with the appropriate transform and by averaging the scans. Regional measurements were calculated by registering a preexisting classified MRI atlas on to the population atlas, which parcellated the brain into a volumetric measurement of 182 different brain regions (Dorr et al., 2008; Richards et al., 2011; Ullmann et al., 2013; Steadman et al., 2014). Subsequently, the deformation of the individual brains to the final atlas space was calculated and analyzed (Nieman et al., 2006) using the Jacobian determinant for each voxel between the genotypes. All statistical analyses were performed in R. We ran a linear model separately for each sex including genotype as contrast. Multiple comparisons were controlled for by using the false discovery rate (FDR) at a stringent setting of 1% (Genovese et al., 2002). FDR limits the expected number of false positives in a set of results to a certain predetermined percentage. For example, if a result is considered significant at a 1% FDR, this means that no more than 1% of the results would be expected to be a false positive. The entire pipeline was made available by the Mouse Imaging Centre in Toronto (^1^ Lerch et al., 2017).

### Immunohistochemistry

Brains were frozen and cut in a cryostat, and 12-μm sections were collected directly on glass slides, at coordinates Bregma 1.10 (anterior brain) and -1.82 (hippocampus), according to the Paxinos and Franklin Mouse Brain Atlas (Paxinos and Franklin, 2001). Glass slides were washed in PBS + Tween 20 0.3% (PBST), 3 min × 5 min. After washing, they were boiled in citrate buffer (Sigma) for 20 min and cooled in ice to room temperature. Slides were then washed again in PBST, 3 min × 5 min, and then incubated in PBST + 2% goat serum (Sigma) (PBSTG) at room temperature for 90 min. Sections were then incubated with antibodies for MBP (MAB386, Merck Millipore; 1:1,000), GFAP (PA1239, Boster Bio, 1:200), or Iba1 (019-19741, Wako Chemical, 1:1,000) diluted in PBSTG, overnight at 4°C. The sections were washed in PBST, 3 min × 5 min, and incubated in H_2_O_2_ 3% for 30 min. After another wash in PBST, slides were incubated with Impress^TM^ HRP anti-rat (MBP; Vector Labs) or EnVision+ system HRP anti-rabbit (GFAP and Iba1; Dako) for 45 min. After this, they were washed once again in PBST for 3 min × 5 min and colored with NovaRED^TM^ solution (Vector Labs). After 7 min, reaction was stopped by adding excess deionized water. Slides were air-dried and mounted with coverslips using Histomount^®^ (National Diagnostics). Due to long-term immersion in fixative supplemented with contrast medium, an elevated level of background was obtained. Digital images of selected fields were acquired in an Olympus microscope equipped with an Olympus digital camera connected to a Dell OptiPlex PC and were displayed using cellSens Standard 1.14 software (Olympus). A 10× objective was used. Digital images were then analyzed with ImageJ. In order to bypass the high amount of background in our images, we devised a method of signal quantification. Briefly, images were transformed from RGB to 8 bit, with a resolution of 2,560 × 1,920 pixels, and mean, median, mode, and standard deviation of the gray values in the entire picture were obtained. With these values, the signal threshold for each picture was determined by subtracting a fixed amount of standard deviations from the mode of the entire picture, which represents the background staining. The number of standard deviations was kept constant within the entire series of pictures for each antibody (2 for MBP, 2.5 for GFAP, and 3 for Iba1). The amount of positive signal marked by the threshold was expressed as the percentage area of total ROI and compared with two-way ANOVA with sex and genotype as factors. In GFAP and Iba1, we estimated the total number of cells, by counting the number of objects (cell bodies) above 250 pixels (**∼**20 μm^2^). The threshold for a significant effect was set at *p* < 0.05. Each animal from each subgroup contributed with only one section per brain area for this analysis.

## Results

### Magnetic Resonance Imaging

**FIGURE 1:**
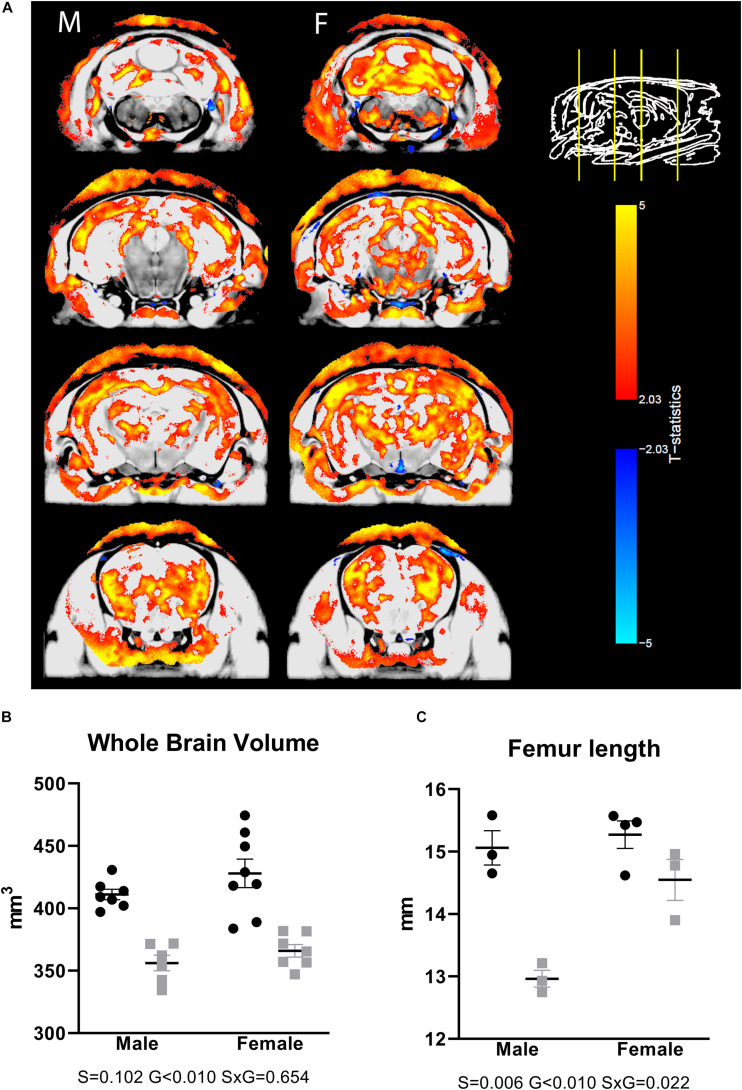
Differences in brain volumes in AdKO_2.0_ mice. **(A)** Coronal MRI slices showing significant differences (FDR 1%) in absolute volume between AdKO_2.0_ mice and age-matched controls. M: male, F: female. The images show coronal MRI slices with the level of significance superimposed. Positive *t*-statistics are shown in red and indicate absolute volume decreases in AdKO_2.0_ mice compared to controls. Negative values are shown in blue and indicate an increase of absolute volume compared to controls. **(B)** Absolute whole-brain volumes. **(C)** Femur lengths for a subgroup of mice. Black dots: wild type. Gray squares: AdKO_2.0_.

To assess whether a CS phenotype results in neuroanatomical changes, we scanned *ex vivo* brain samples using high-resolution MRI. We observed volumetric differences that were widespread over the brain, including both gray and white matter areas (Figure 1A), with prominent differences, particularly in the corpus callosum and large parts of the cortex. Looking at absolute volumes, we found a significantly reduced brain size in males, with a trend toward significance in females (Figure 1B). For a subgroup of animals, the femur length was measured, showing a significantly smaller value in the transgenic animals (Figure 1C), indicating that the large difference in brain volume between WT and transgenic mice may be due to generalized growth retardation and not brain specific. Therefore, we also normalized the segmented structures for the whole-brain volume. Relative volume differences were then apparent for a number of brain regions. Supplementary Table 1 provides an overview of all brain structures with differential relative volumes and uncorrected significance level. A list of all segmented structures with absolute and relative volumes and uncorrected *p*-values is given in Supplementary Table 2.

**FIGURE 2:**
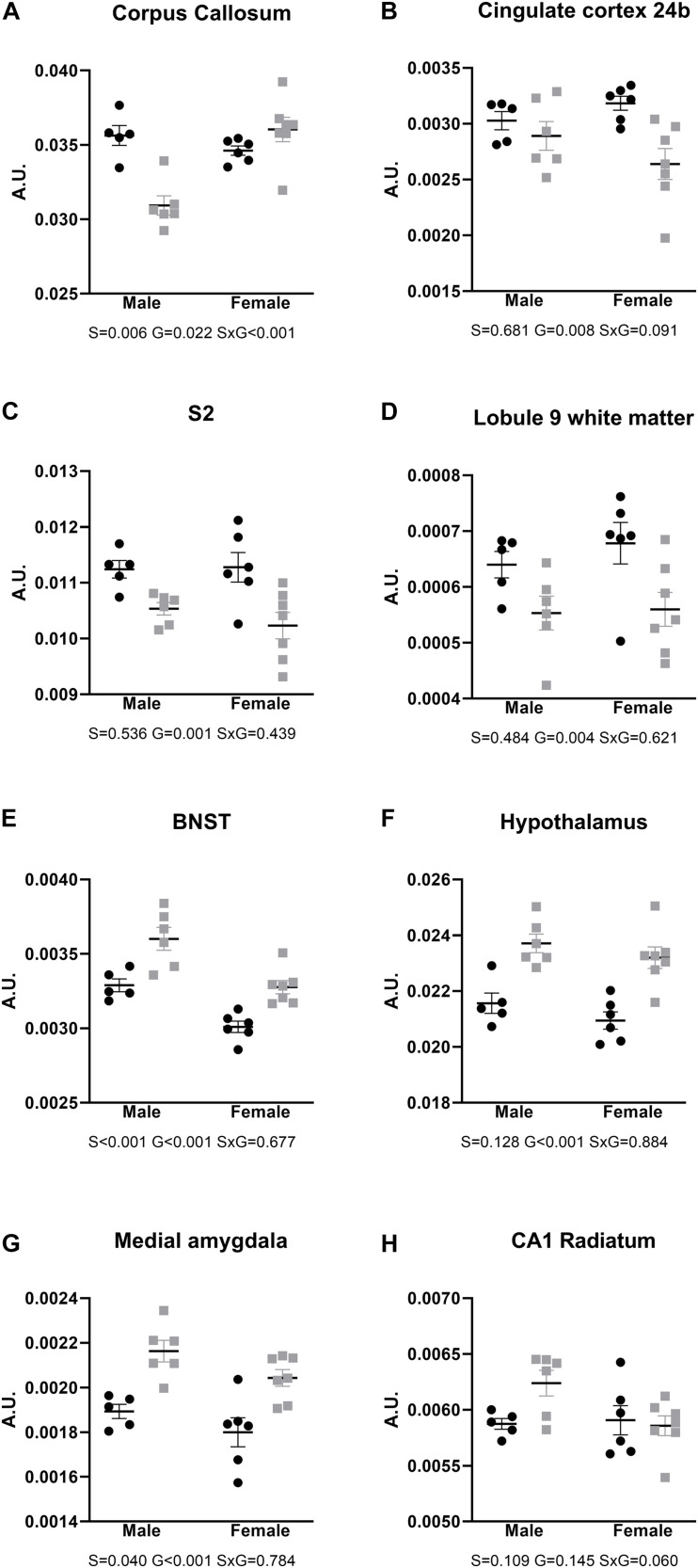
Relative volumes of brain areas and regions, compared with two-way ANOVA. **(A–D)** Areas with smaller relative brain volumes in the AdKO_2.0_ mice. **(E–G)** Areas with bigger relative brain volumes in the AdKO_2.0_ mice. **(H)** CA1 s. radiatum as representative for the hippocampus. S2: secondary somatosensory cortex; BNST: bed nucleus of the stria terminalis; CA1: cornu ammonis 1. S, G, and S × G: *p* value of the source of variation (sex, genotype, and interaction, respectively). Black dots: wild type. Gray squares: AdKO_2.0_. AU, arbitrary units.

After FDR correction for multiple comparisons, the corpus callosum showed a robust significant difference in males, with a significantly smaller relative volume in AdKO_2.0_ males than in WT males (Figure 2A). Lower volumes were also observed in frontal cortical areas, independent of sex, as well as cerebellar white matter (Figures 2B–D). In contrast, the bed nucleus of the stria terminalis, hypothalamus, and medial amygdala were found to be larger in AdKO_2.0_ mice brain (Figures 2E–G). Finally, no hippocampal structures differed in relative volumes between genotypes; although the CA1 stratum radiatum appeared larger in male AdKO_2.0_ mice compared to WT male counterparts in the pairwise comparisons available in the MRI pipeline. However, subsequent two-way ANOVA showed no significant effects for factors sex and genotype or their interaction (Figure 2H).

### Immunohistochemistry

**FIGURE 3:**
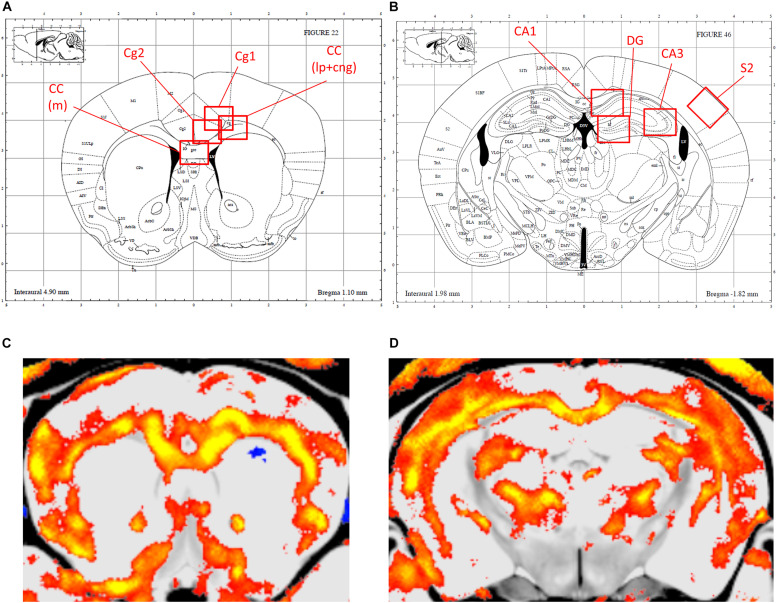
Localization of the areas that were analyzed for immunohistochemical staining. **(A,B)** Indications of the regions of interest (ROIs) in the corresponding levels of the Paxinos atlas for the anterior brain (Bregma 0.98–0.86 mm), and dorsal hippocampus (Bregma -1.82 to -1.94) (Reprinted with permission by Elsevier Ltd.). **(C,D)** orresponding MR images, with smaller volumes indicated in color. Cg1, cingulate cortex 1; Cg2, cingulate cortex 2; CC (lp), corpus callosum lateroproximal; CC (m), corpus callosum medial; S2, secondary somatosensory cortex; CA1, cornu ammonis 1; CA3, cornu ammonis 3.

**FIGURE 4:**
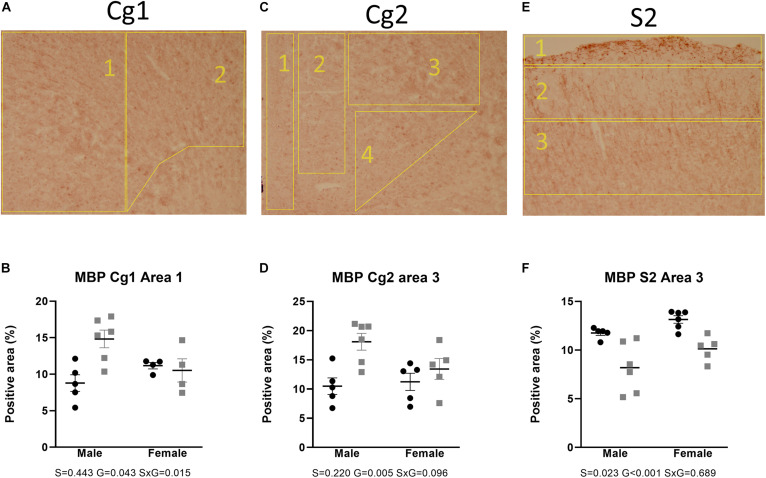
Staining and quantification of MBP at the anterior level. Numbered polygons in stained sections indicate neuroanatomical parcellations. **(A–D)** In the cingulate cortex (Cg), AdKO_2.0_ mice had a male-specific *increase* in MBP-positive area compared to WT mice. **(E,F)** In the somatosensory cortex (S2), there was a reduction in MBP-immunopositive area in AdKO_2.0_ mice of both sexes. S, G, and S × G: *p* value of the source of variation (sex, genotype, and interaction, respectively). Black dots: wild type. Gray squares: AdKO_2.0_.

**FIGURE 5:**
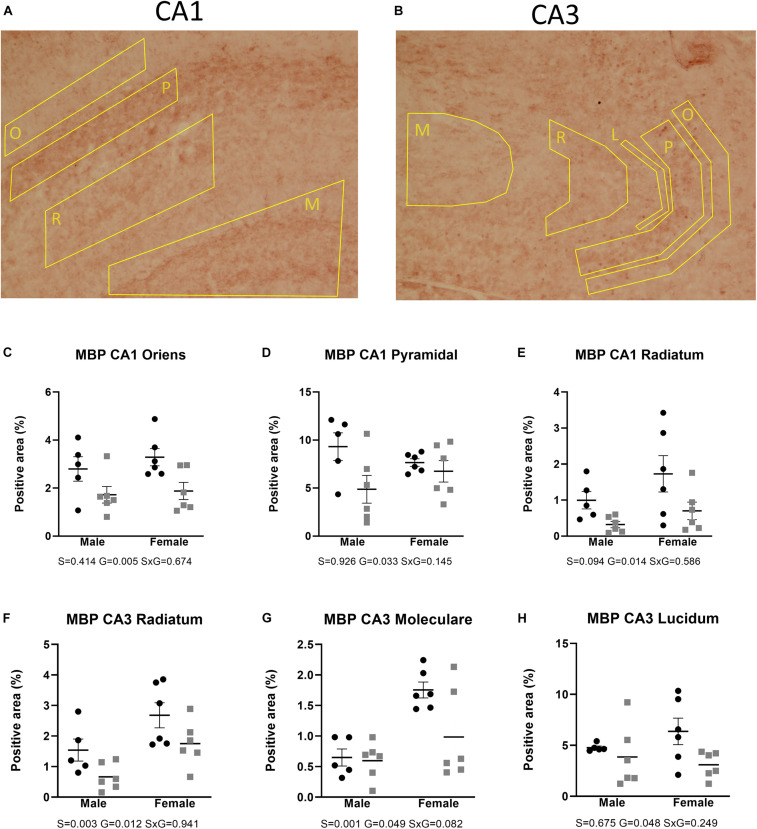
Expression of MBP in the dorsal hippocampus. Coded polygons in stained sections indicate neuroanatomical parcellations. **(A,C–E)** In CA1, AdKO_2.0_ mice showed a decrease in MBP-positive areas in both sexes in oriens (O) and radiatum (R) strata and a male-specific decrease in the pyramidal layer, compared to WT mice. **(B,F)** In CA3, AdKO_2.0_ mice showed a decrease compared to WT mice in expression in both sexes in the s. radiatum. **(G,H)** In the s. moleculare (M) and s. lucidum (L), a female-specific decrease was shown. S, G, and S × G: *p* value of the source of variation (sex, genotype, and interaction, respectively). Black dots: wild type. Gray squares: AdKO_2.0_.

Figures 2, 3 show the relative volumetric differences between the brain structures that were chosen for immunohistochemistry, selected on prior data in active disease (hippocampus) (Starkman et al., 1992, 1999; Burkhardt et al., 2015), long-lasting effects in CD patients (Cg) (Andela et al., 2013), and reduced volumes in the present MRI analysis (S2). As mentioned before, we obtained a high level of background in our preparations due to long-term immersion in fixative supplemented with contrast medium, which we corrected for by using our thresholding method. MBP as a marker for oligodendrocyte function showed a main effect for genotype in cingulate cortex areas (Cg1 *p* = 0.043, Cg2 *p* = 0.005), indicating increased immunoreactivity in the AdKO_2.0_ mice. For the Cg1 area, this effect was male specific (interaction effect *p* = 0.015). This interaction between sex and genotype was present at the trend level of the Cg2 area (*p* < 0.1) (Figure 4). At the hippocampal level, however, there was decreased MBP immunoreactivity in the CA1 and CA3 fields (CA1: s. oriens *p* = 0.005, s. pyramidalis *p* = 0.033, s. radiatum *p* = 0.014; CA3: s. lucidum *p* = 0.048, s. radiatum *p* = 0.012) (Figure 5). At this frontocaudal level, a decrease of MBP signal in AdKO_2.0_ mice was also observed in the S2 cortex (main effect *p* < 0.001) (Figure 5). MBP signal showed a main effect of sex in the hippocampal CA3 area (CA1: s. moleculare *p* = 0.047; CA3: s. radiatum *p* = 0.003, s. moleculare *p* = 0.001) and S2 cortical area (area 3 *p* = 0.023), reflecting higher immunoreactivity in female mice. Overall, at the hippocampal level and somatosensory cortex, MBP immunohistochemistry was reduced in AdKO_2.0_ mice, while in the cingulate cortex, there was a male-specific increase. All significant effects are listed in Supplementary Table 3A.

**FIGURE 6:**
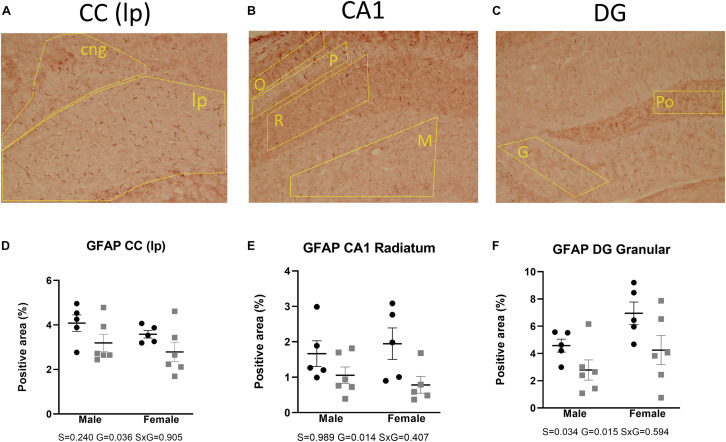
Expression of GFAP in the corpus callosum **(A)**, hippocampal CA1 area **(B)**, and DG **(C)**. Coded polygons in stained sections indicate neuroanatomical parcellations. In the three regions, reduced immunostaining in the AdKO_2.0_ mice of both sexes was observed compared to that in WT mice **(D–F)**. S, G, and S × G: *p* value of the source of variation (sex, genotype, and interaction, respectively). Black dots: wild type. Gray squares: AdKO_2.0_.

GFAP immunohistochemistry was used as a reactive astrocytic marker. Two-way ANOVA tests showed a main effect of genotype, reflecting a decrease of immunoreactivity in the AdKO_2.0_ mice throughout the brain regions. This was found in the corpus callosum (*p* = 0.036) and in the hippocampal CA1 (s. radiatum *p* = 0.014) and dentate gyrus (DG; granular layer *p* = 0.015) (Figure 6). Also, a main effect of sex was found in CA1 (s. moleculare *p* < 0.001), CA3 (*p* = 0.030), and DG (granular layer *p* = 0.034), which indicates a higher expression in females. The number of GFAP-positive cells was not different between genotypes (Supplementary Table 4A). Overall, in both frontocaudal levels, GFAP staining was diminished in ADKO_2.0_ mice. All significant effects are listed in Supplementary Table 3B.

**FIGURE 7:**
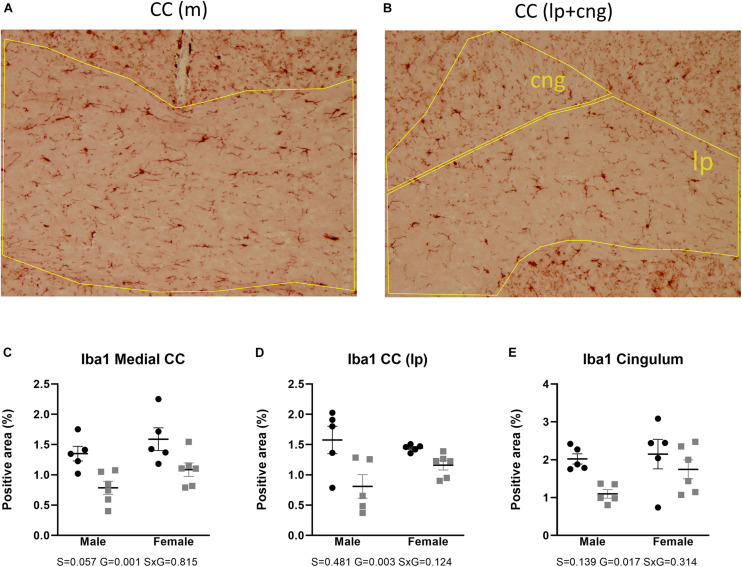
Expression of Iba1 in the corpus callosum (medial and lateroproximal) **(A,B)**. Coded polygons in stained sections indicate neuroanatomical parcellations. In medial and lateroproximal (lp) areas, a decrease in expression in AdKO_2.0_ mice compared to that in WT mice was observed **(C,D)**. In the cingulum (cng), there was a male-specific decrease in the AdKO_2.0_ mice **(E)**. S, G, and S × G: *p* value of the source of variation (sex, genotype, and interaction, respectively). Black dots: wild type. Gray squares: AdKO_2.0_.

**FIGURE 8:**
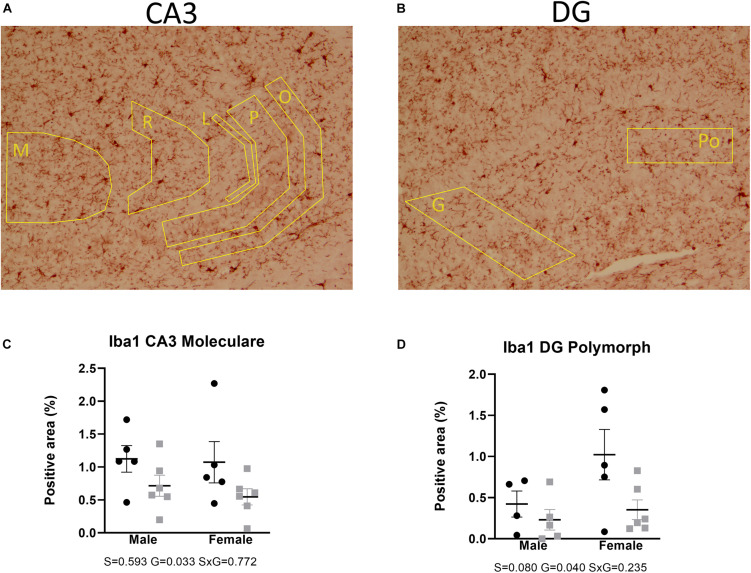
Expression of Iba1 in the hippocampal CA3 **(A)** and DG **(B)**. Coded polygons in stained sections indicate neuroanatomical parcellations. In the stratum moleculare (M) of CA3, AdKO_2.0_ of both sexes showed a decrease in expression, compared to WT mice **(C)**. In the polymorph (Po) layer of DG, there is a female-specific decrease in AdKO_2.0_ mice **(D)**. Data were analyzed by two-way ANOVA, α: 0.05. S, G, and S × G: *p* value of the source of variation (sex, genotype, and interaction, respectively). Black dots: wild type. Gray squares: AdKO_2.0_.

Microglial activity was assessed by means of Iba1 immunohistochemistry. Two-way ANOVA showed that Iba1 immunoreactivity in the anterior brain of the AdKO_2.0_ mice was significantly decreased in the corpus callosum (medial *p* = 0.001, lateroproximal *p* = 0.003, cingulate *p* = 0.017) (Figure 7). Likewise, at the hippocampal level, decreases were also observed in CA3 (s. moleculare *p* = 0.033) and DG (polymorph *p* = 0.040) (Figure 8) in AdKO_2.0_ mouse brains. There were no effects of sex or interaction. In the corpus callosum, we observed a reduced total number of Iba1-positive cells (Supplementary Table 4B). Overall, in both frontocaudal levels, Iba1 staining was diminished in ADKO_2.0_ mice. All significant effects are listed in Supplementary Table 3C.

## Discussion

The present study used ADKO_2.0_ mice to study the consequences of long-term GC exposure for the brain. The AdKO_2.0_ mice had decreased relative volumes in a number of anterior cortical brain structures (cingulate cortex and somatosensory cortex) and in a number of white matter structures (corpus callosum, but also cerebellar white matter tracts). In contrast, we observed higher relative volumes in the medial amygdala, BNST, and hypothalamus. Markers for non-neuronal cell types showed that there were bidirectional effects on MBP, that is, a male-specific increase in immunoreactive area in the cingulate cortex and a sex-independent decrease in the somatosensory cortex and the hippocampus. Activated astrocyte and microglial markers GFAP and Iba1 were suppressed at both the anterior and hippocampal levels independent of sex.

Our analysis was based in one section per brain area per animal. This may increase technical variation, which would increase the chance of a false-negative result. For most markers, data provided clear indication of widespread changes, and we may underestimate the number of brain areas in which they might occur.

The volumetric changes found in the AdKO_2.0_ mouse brains were extensive and widespread. In principle, the whole-brain volume reduction reflects previous findings in human studies during active CS. Both pediatric and adult CS patients have a decrease of total brain volume (Bourdeau et al., 2002; Merke et al., 2005). In the analysis of individual brain areas, our results are in line with human studies that found changes in cerebellum and cortex volumes (including insular and cingulate regions) but found no effects when measuring the hippocampus or amygdala (Merke et al., 2005; Santos et al., 2014; Jiang et al., 2017a, b; Hou et al., 2020). However, other studies reported decreases in hippocampal volume (Starkman et al., 1992, 1999; Burkhardt et al., 2015), and this was also observed after chronic exogenous GC exposure (Zhang et al., 2015). In the AdKO_2.0_ brains, volumetric decreases were prominent in white matter areas, such as the corpus callosum and cingulate cingulum. In human studies, patients show smaller absolute volumes of white matter (Jiang et al., 2019; Tirosh et al., 2020), as well as reduced fractional anisotropy and increased mean diffusivity, which indicate white matter deterioration (Pires et al., 2015).

The overall parallels between the data from human imaging studies and ours indicated that the AdKO_2.0_ is an adequate model of CS effects on the brain and opened the opportunity to study the origins of such changes in further detail. It was particularly interesting that the corpus callosum was smaller than in wild-type mice, because this region has been described to still be affected in long-term remission (Andela et al., 2013; van der Werff et al., 2014). We decided therefore to focus our analyses on glial cells in areas that showed reduced relative volumes. We also included the hippocampus, given the extensive literature on its response to GCs.

We observed a decrease in MBP expression in the hippocampus, which may relate to the long-term loss of white matter integrity in remitted CS patients. We did not acquire diffusion tensor imaging (DTI) data in our mice; thus, a direct comparison is not possible. A body of literature suggests inhibiting effects of GCs on oligodendrocyte function and proliferation (Miguel-Hidalgo et al., 2019; hippocampus and pyriform cortex) and white matter (Dunlop et al., 1997; Alonso, 2000; Quinlivan et al., 2000). Work in multiple sclerosis models also supports negative effects of GCs on MBP expression and white matter (Sieve et al., 2004; Chari et al., 2006). The reduced MBP expression seems a logical correlation to the reduced white matter volume in the MRI data of our mice. However, the literature also reports protective effects of GCs, mainly in oligodendrocyte cultures (Kumar et al., 1989; Byravan and Campagnoni, 1994; Lee et al., 2008; Xu et al., 2009). A protective role was also observed in *in vivo* settings (Raschke et al., 2008; Sun et al., 2010). The contradictory literature on GC effects on MBP expression may reflect a biphasic effect, with low doses stimulating and high doses being detrimental (Almazan et al., 1986).

MBP immunoreactivity was higher in parts of the prefrontal cortex of AdKO_2.0_ mice, in apparent contrast with the lower volume observed in both active and remitted patients (Andela et al., 2013; Hou et al., 2020), as well as in our mice. The increased MBP immunoreactive surface area in conjunction with lower volume might be due to a different myelin organization, perhaps a more open myelin conformation (“loose myelin”). In fact, it has been observed that myelin lamellae fail to associate and compact after chronic GC treatment (7 days) in rats (Chari, 2014). Overall, our data suggest that in the mouse brain, this cingulate cortex has a particular sensitivity to long-term GC exposure.

The decrease in expression of GFAP is also in line with previous reports on the effects of GCs on astrocytes. It has been shown in other studies that GCs, e.g., corticosterone and dexamethasone, decreased cell viability and proliferation in astrocyte cultures and GFAP expression *in vivo* (Unemura et al., 2012; Guo et al., 2013; Zhang et al., 2015; Freitas et al., 2016). Similarly, chronic stress has been shown to decrease astrocytic cell size, count, and process length in the hippocampus and prelimbic cortex (Czeh et al., 2006; Banasr and Duman, 2008). Chronic stress also decreased expression of GFAP in the hippocampus and brainstem (Ye et al., 2011; Imbe et al., 2013; Lou et al., 2018). However, in some experimental settings, stressors and GCs led to increased astrocyte activation, in a GR-dependent manner (Revsin et al., 2009; Shields et al., 2012; Zia et al., 2015; Huet-Bello et al., 2017). It is well-known that GC effects can be context dependent (Meijer et al., 2019). Our data are in line with the notion that in the adult brain, in the absence of overt stressors or pathology, GCs suppress astrocyte activity.

Microglial immunoreactivity was clearly lower in the AdKO_2.0_ mice, an effect that is in line with a general immunosuppressive effects of chronically elevated GCs. In agreement with our data, previous works have described that microglia shrink and stop proliferating when corticosterone is added to microglial cultures (Tanaka et al., 1997; van Olst et al., 2018). Cell viability was compromised due to a cytotoxic effect of GCs, and this effect could be blocked by mifepristone (Nakatani et al., 2012; Cerqueira et al., 2013). Chronic corticosterone administration induces retraction of microglial processes in the hippocampus of mice (Park et al., 2011; Freitas et al., 2016), and adrenalectomy greatly increases microglial activation in the hippocampus and hypothalamus after acute stress; this response is ablated by exogenous corticosterone administration in rats (Sugama et al., 2013). In models of neuronal damage, the same trend has been observed for synthetic GCs (methylprednisolone and triamcinolone) (Schroter et al., 2009; Li et al., 2011).

A number of reports, however, described different effects. GCs may potentiate microglial responses *via* a priming mechanism, concomitant with an increase of Iba1 but suppression of cytokine production (Frank et al., 2014). Moreover, chronic restraint stress paradigms increased Iba1 immunoreactivity in various areas of the brain, including the anterior cingulate cortex and hippocampus (Tynan et al., 2010; Hinwood et al., 2012) and stimulated microglial process lengthening and branching out (Hinwood et al., 2013). However, this effect was observed only in large cells, and there might be a timing factor involved, as it has been also observed that induction of microglial proliferation by stress is time limited (Nair and Bonneau, 2006). The conflicting data may be resolved by taking time of exposure into account, since some studies showed stimulatory effect of short-term exposure but inhibitory effects of long-term exposure (Kreisel et al., 2014; Winkler et al., 2017). Our mutant mouse data are compatible with the latter, as they had been exposed to chronic hypercorticosteronemia (8–10 weeks).

The diversity of effects of GCs and stress on brain volume and on cell morphology might be related to age, duration of treatment, sex, stress context, or type of GC molecule, as synthetic GCs may differ from endogenous steroids in some of their effects (Meijer and de Kloet, 2017). Our model closely resembles the conditions of endogenous GC excess in humans in terms of physiological traits and, particularly, the time course of the endocrine imbalance. It remains to be determined to what extent the observed effects depend on direct activation of brain GRs and to what extent the context of, for example, changed metabolism-related factors contributes to the observed effects, as it is known that GR activity is also influenced by its association to (tissue-specific) coregulators and other transcription factors (Spaanderman et al., 2018; Viho et al., 2019). Thus, further studies are necessary to identify specific transcriptional changes that are associated with, and perhaps causal for, the changes in brain morphology that are apparent in these mice.

The present work is, to our knowledge, the first translational study to assess brain volumetric differences together with alterations in glial cell markers. In both aspects, we found significant changes in the selected brain regions. The hippocampus and prefrontal cortex are key regions in the brain for cognitive processing and integration, respectively. In human studies in CS patients both with active disease and in remission, various degrees of cognitive deficit are found. Cognitive performance has been related to myelin integrity, particularly in the hippocampus and prefrontal cortex (Nickel and Gu, 2018). In a similar manner, microglial morphological changes in the prefrontal cortex have been reported in obesity-related cognitive impaired rats (Bocarsly et al., 2015). We consider that our present results provide an opportunity to contribute to the study of participation of glial cells in cognition; thus, in the future, a number of neurobehavioral assays should be tested in the AdKO mice.

## Data Availability Statement

The original contributions presented in the study are included in the article/Supplementary Material, further inquiries can be directed to the corresponding author/s.

## Ethics Statement

The animal study was reviewed and approved by C2E2A (Comité d’Ethique pour l’Expérimentation Animale en Auvergne), Commission Nationale de Réflexion Ethique sur l’Expérimentation Animale, and Ministère français de l’Enseignement supérieur, de la Recherche et de l’Innovation. République Française.

## Author Contributions

JA, LW, and OM designed experiment. IS-B, TD, and NM generated mouse models, managed mouse cohorts and tissues preparation for MRI, and histological analyses. GP and CK conducted MRI measurements. ES and LW analyzed MRI data. JA performed histological procedures and analyzed histological data. JA, LW, AP, AM, and OM prepared manuscript. LW, AM, and OM supervised the project. All authors contributed to the article and approved the submitted version.

## Conflict of Interest

The authors declare that the research was conducted in the absence of any commercial or financial relationships that could be construed as a potential conflict of interest.

## Footnotes

1 https://github.com/Mouse-Imaging-Centre
